# Autonomic dysfunction as the initial presentation in spinocerebellar ataxia type 3: A case report and review of the literature

**DOI:** 10.3389/fneur.2022.967293

**Published:** 2022-09-27

**Authors:** Yi Jin, Yuchao Chen, Dan Li, Mengqiu Qiu, Menglu Zhou, Zhouyao Hu, Qiusi Cai, Xulin Weng, Xiaodong Lu, Bin Wu

**Affiliations:** ^1^Department of Neurology, The Affiliated Hospital of Hangzhou Normal University, Hangzhou, China; ^2^School of Clinical Medicine, Hangzhou Normal University, Hangzhou, China; ^3^Translational Medicine Center, The Affiliated Hospital of Hangzhou Normal University, Hangzhou, China

**Keywords:** spinocerebellar ataxia type 3, early stage, initial presentation, sexual dysfunction, autonomic dysfunction

## Abstract

Spinocerebellar ataxia type 3 (SCA3), as the most frequent autosomal dominant ataxia worldwide, is characterized by progressive cerebellar ataxia, dysarthria and extrapyramidal signs. Additionally, autonomic dysfunction, as a common clinical symptom, present in the later stage of SCA3. Here, we report a 44-year-old male patient with early feature of autonomic dysfunction includes hyperhidrosis and sexual dysfunction, followed by mild ataxia symptoms. The Unified Multiple System Atrophy Rating Scale (UMSARS) indicated significant dysautonomia during autonomic function testing. Combination of early and autonomic abnormalities and ataxia would be more characteristic of the cerebellar type of multiple system atrophy (MSA-C), the patient's positive family history and identification of an *ATXN3* gene mutation supported SCA3 diagnosis. To best of our knowledge, the feature as the initial presentation in SCA3 has not been described. Our study demonstrated that autonomic dysfunction may have occurred during the early stages of SCA3 disease.

## Introduction

Spinocerebellar ataxia type 3 (SCA3), also known as Machado–Joseph disease (MJD), is the most common autosomal dominant hereditary spinocerebellar disease. It was caused by an abnormal CAG repeat expansion at exon 10 of *ATXN3* gene (1). Clinical features of MJD are heterogeneous. The characteristic manifestations such as progressive cerebellar ataxia, dysarthria, saccade slowing, and peripheral neuropathy have been observed, non-characteristic features, such as sleep disorders, cognitive, and psychiatric disturbances have also been observed (2).

Autonomic abnormalities are well recognized in Parkinson's disease (PD) and MSA-C, and often found in the early stage of disease (3). The autonomic dysfunction was a common symptom in SCA3 type and was reported in different racial groups (4). Compared with motor symptoms in SCA3 patients, autonomic dysfunction often presented in a later stage of disease (4). Among various manifestations of autonomic dysfunctions, voiding problems and thermoregulatory disturbance were the most prevalent in SCA3 patients with dysautonomia (5). Here, we present clinical and genetic findings of a Chinese patient who sought treatment for isolated autonomic failure in the early stage of disease and later developed ataxia and genetically proven SCA3.

## Case presentation

**Figure 1:**
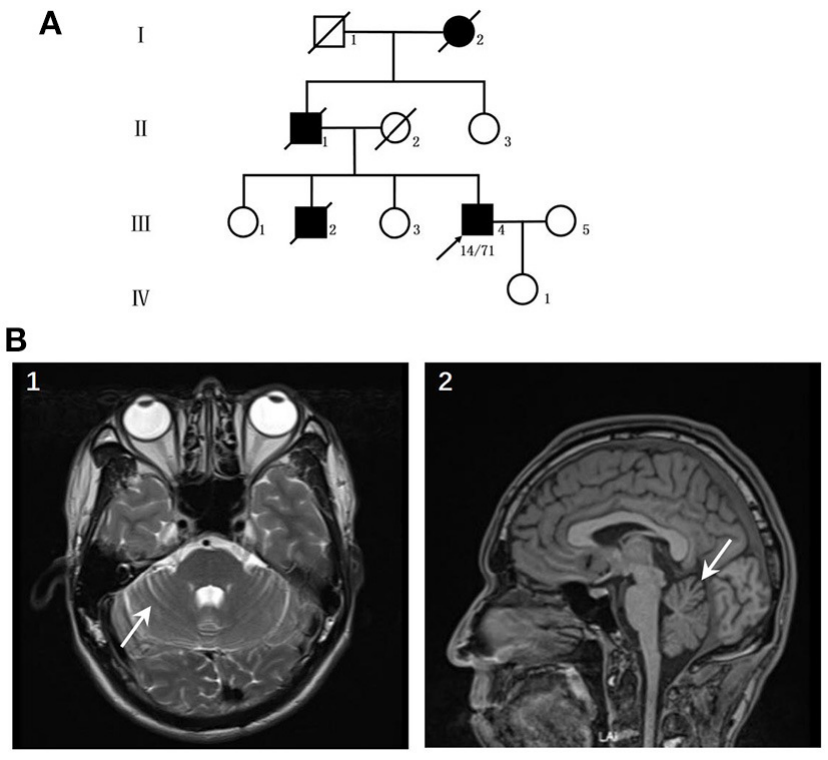
**(A)** Pedigree of the patient's family. **(B)** Neuroimaging features of the patients. (1–2) patient's MRI revealed prominent cerebellar atrophy (arrow).

The patient was a 44-year-old male who suffered from hyperhidrosis, heat intolerance and sexual dysfunction since the age of 37. Over the next year, he developed difficulty emptying his bladder and erectile failure without any motor features. The patient complained of these symptoms and sought medical advice from the department of urology. He was treated with anti-sexual dysfunction therapy, found to be ineffective during the brief trial period. At the age of 39, he developed gait problems and had difficulty in running. He noticed that his father and older brother had similar gait symptoms. In addition, his older brother suffered from the severely ataxia symptoms and suicided at the age of 25. He was seen in our clinic for the first time 3 years later, with a 3-years history of progressive ataxia symptoms. Neurological examinations revealed that both limbs had MRC grade 5 muscle strength. All four extremities had increased muscle tone, but deep tendon reflexes remained intact. The sensation was normal. Babinski's signs were negative bilaterally. In the coordination examination, he presented mild cerebellar ataxia of limb and trunk. Bilateral finger-nose, rotation tests, and heel-knee-tibia tests were mild awkward. Romberg sign could be checked for ability to stand. He scored 22/100 for the International Cooperative Ataxia Rating Scale (ICARS), the total score of Scale for the Assessment and Rating of Ataxia (SARA) was 7.5/40. In the autonomic function study, autonomic symptoms were evaluated using the Unified Multiple System Atrophy Rating Scale (UMSARS) and Scales for Outcomes in Parkinson's Disease - Autonomic (SCOPA-AUT), the total score of UMSARS and SCOPA-AUT (Parts I + II) were 11/69 and 15/104, respectively. The affective and cognitive components of SCA3 were evaluated using the Hamilton Anxiety Scale (HAMA), 24-item Hamilton Rating Scale for Depression (HAMD-24) and Mini-Mental State Examination (MMSE). The total score of HAMA, HAMD-24 and MMSE were 13/56, 6/83, and 28/30, respectively. Serum tests were normal. Brain magnetic resonance imaging (MRI) revealed prominent cerebellar atrophy (Figure 1). Genetic testing showed 14/71 CAG repeats in *ATXN3* gene. After follow-up for 16 months later, he developed early satiety, blurred vision and nocturia. A neurological examination presented mild limbs and trunk ataxia. The total scores of ICARS, SARA, UMSARS and SCOPA-AUT (Parts I + II) were 24/100, 4.5/40, 15/69, and 18/104, respectively.

**Figure 2:**
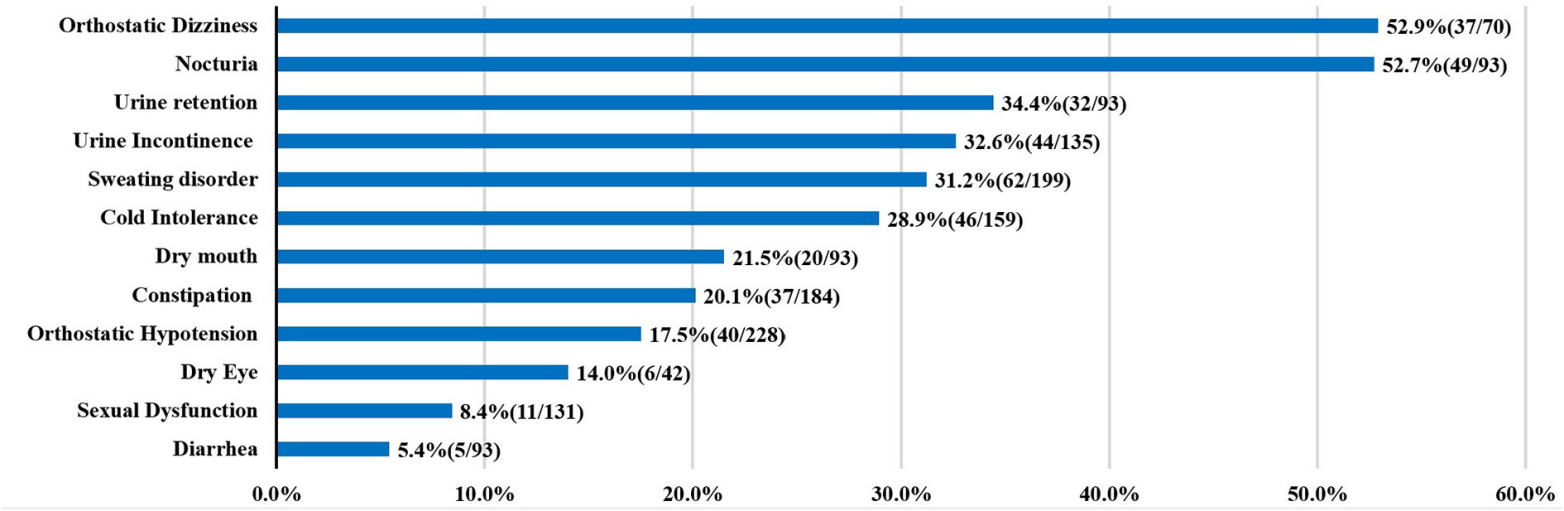
Autonomic dysfunctions of SCA3. For each clinical manifestation of autonomic dysfunction, the proportion of patients is indicated.

Up to now, the publications about autonomic dysfunction and SCA3 were all small sample researches, the results about the frequency of dysautonomia cannot be considered definitive. Here, we summarized the clinical factors and autonomic dysfunction from individual reports (Table 1) (5–15). In the prior studies orthostatic dizziness and urinary disturbance were common autonomic features, followed by sweating disorder and cold intolerance (Figure 2).

**Table 1:** Clinical factors and autonomic dysfunction from published studies.

**References**	**Sample size**	**Gender M/F**	**Countries**	**AAO (years)**	**Age (years)**	**Duration of disease (years)**	**CAG repeats**	**Orthostatic dizziness**	**Orthostatic hypotension**	**Urinary disturbance**			**Sexual dysfunction**	**Constipation **	**Diarrhea **	**Cold intolerance**	**Sweating disorder**	**Dry mouth**	**Dry eye**
										**Nocturia**	**Urineincontinence **	**Urine retention**							
Hirayama et al. (6)	66	–	Japan	30.7 ± 12.4	–	13.2 ± 8.0	–	–	10	21			2	4	–	0	4	–	–
Schöls et al. (7)	42	19/23	Germany	37.5 ± 9.9	–	10.1 ± 6.1	74.0 ± 3.5	–	–	–	–	–	–	–	–	–	–	–	–
Watanabe et al. (8)	20	7/13	Japan	35.4 ± 11.0	–	11.4 ± 5.4	72.2 ± 3.1	–	–	11			–	–	–	–	–	–	–
Kazuta et al. (9)	19	6/13	Japan	–	55.7 ± 10.9	13.8 ± 5.5	–	–	5	–	–	–	–	–	–	–	–	–	–
Yeh et al. (5)	15	4/11	China	29.9 ± 10.3	40.2 ± 13.2	10.3 ± 6.9	76.3 ± 3.9	7	2	8	2	–	1	2	1	8	–	5	5
Asahina et al. (10)	10	4/6	Japan	–	55 ± 16	11.5 ± 8.5	65.9 ± 6.2	–	1	4			–	2	–	–	–	–	–
França et al. (11)	50	30/20	Portugal/ Brazil	35.5(3–55)	46.5 (10–73)	11.2(2–46)	72 (65–81)	–	2	32	23	27	8	15	4	24	24	8	–
Yamanaka et al. (12)	15	7/8	Japan	–	48.9 ± 15.1	11.5 ± 7.7	68.6 ± 5.5	4	–	–	–	4	–	6	–		5	–	–
Takazaki et al. (13)	40	17/23	Brazil	–	46.3 ± 12.5	8.9 ± 5.2	67 ± 5	26	10	–	–	–	–	–	–	–	18	–	–
Moro et al. (14)	28	11/17	Brazil	36.4 ± 7.8	49.7 ± 9.7	13.5 ± 7.3	70 (67–75)	–	10	9	11	1	–	8	0	14	11	7	1
Jang et al. (15)	26	–	–	–	–	–	–	–	–	2			–	–	–	–	–	–	–

AAO, age at onset; M, male; F, female; (–): not available.

## Discussion

SCA3 is a hereditary and clinically heterogeneous disease characterized by characteristic limb ataxia and various dystonia combinations, oculomotor disturbance, sleep disorder, and autonomic dysfunction. And gait disturbance is a common symptom in SCA3 patients, usually presented in the early stage of disease. Additionally, other atypical features developed following the onset of ataxia.

In this study, we report a SCA3 patient with initial symptoms of severely autonomic dysfunction, followed by mild unsteady gait. UMSARS and SCOPA-AUT were used to monitor the patient, indicating obvious autonomic dysfunctions. According to the patient, his cousin had prominent limb ataxia problems, whereas preserved autonomic function. Our results further confirm the clinical heterogeneity in SCA3. Additional factors, such as environmental or gene modifiers may be associated with variable phenotypes of SCA3.

In the early stage of disease, our patient complained of isolated prominent erectile failure and hyperhidrosis lacking-ataxia, which prompted him to seek medical treatment from an unrelated department. Additionally, the combination of early severe dysautonomia and limb ataxia is generally typical of MSA-C (4). The clinical features of SCAs with early autonomic dysfunction can be easily confused with MSA-C in the absence of clear family history. Typical family history was present in our patient, and genetic analysis revealed the repeats CAG expansion of *ATXN3*, supporting SCA3 diagnosis. Interestingly, a patient with clinical features consistent with MSA-C and a SCA3 mutation but pathologically confirmed MSA-C had been described, implying that abnormal *ATXN3* gene expansion may be a risk factor for the development of MSA-C (16). This might explain the occurrence of MSA-like clinical features such as dysautonomia in SCA3 patients. Until now no association between MSA and CAG repeat sizes in *ATXN3* were observed in a study of 200 subjects with a clinical diagnosis of MSA, possibly due to sample size (17). As a result, additional studies with larger sample sizes might help to fully explain the association between pathological MSA features and SCA3 mutation.

The specific mechanism of sexual dysfunction in SCA3 is debated. Onuf's nucleus and intermediolateral (IML) column, both contain neurons for autonomic function, and play a crucial role in erection. Onuf's nucleus involvement has been found in PD and MSA patients (18), while IML nuclear involvement is common in MSA (19). As demonstrated in animal model, many neuronal populations of the CNS are involved in the supraspinal control of micturition, defecation and sexual functions (20). We reasoned that the patient's bladder and erectile dysfunction may be partly influenced by CNS lesions, although a coexisting peripheral neuropathy might contribute to dysautonomia in our patient. Unfortunately, he refused to undergo EMG studies. Recently, a study about MSA found that patients with low pyramidal scores are more likely to have erectile dysfunction (21). The association between ataxia symptoms and autonomic dysfunction in SCA3 requires future exploration.

Our results indicate that sexual dysfunction may be the initial symptom in SCA3, but may often misdiagnosed, so clinicians should attention to the differential diagnosis in daily clinical practice. Autonomic dysfunction as well as limb ataxia would seriously affect patient's quality of life and emotions, clinicians should focuse on the non-characteristic features as well as characteristic features. The treatment requires a multidisciplinary approach, to maximize function and reduce complications, and this is of prime importance for SCA3 patients.

## Conclusion

In summary, we described a SCA3 patient who developed severe dysautonomia at an early age and was later diagnosed with mild ataxia. Our case demonstrates that the clinical spectrum of SCA3 may be broader than previously believed, encompassing the presence of severe and disabling autonomic dysfunction since the early stage of disease.

## Data availability statement

The original contributions presented in the study are included in the article/supplementary material, further inquiries can be directed to the corresponding authors.

## Ethics statement

The studies involving human participants were reviewed and approved by the Ethical Committee of the Affiliated Hospital of Hangzhou Normal University. The patients/participants provided their written informed consent to participate in this study. Written informed consent was obtained from the individual(s) for the publication of any potentially identifiable images or data included in this article.

## Author contributions

YJ and YC designed the work. YJ, YC, DL, MQ, MZ, ZH, QC, XW, XL, and BW initiated the project, collected, and analyzed the data. YJ wrote the manuscript. BW commented and revised on the manuscript. YC and XL supervised all aspects of the project. All authors read and approved the final manuscript.

## Funding

This study was supported by the Medical and Health Science and Technology Major Project of Hangzhou to XL (ZD20200049).

## Conflict of interest

The authors declare that the research was conducted in the absence of any commercial or financial relationships that could be construed as a potential conflict of interest.

## Publisher's note

All claims expressed in this article are solely those of the authors and do not necessarily represent those of their affiliated organizations, or those of the publisher, the editors and the reviewers. Any product that may be evaluated in this article, or claim that may be made by its manufacturer, is not guaranteed or endorsed by the publisher.
